# Remission, cognition and functioning in patients with schizophrenia: a systematic review

**DOI:** 10.1590/1980-5764-DN-2025-0296

**Published:** 2025-09-19

**Authors:** Edmundo Rinolino Magalhães Flores, Breno Fiuza Cruz, Lucas Machado Mantovani, João Vinicius Salgado

**Affiliations:** 1 Universidade Federal de Minas Gerais, Departamento de Morfologia, Belo Horizonte MG, Brazil.; 2 Universidade Federal de Minas Gerais, Departamento de Saúde Mental, Belo Horizonte MG, Brazil.; 3 Fundação Hospitalar do Estado de Minas Gerais, Instituto Raul Soares, Belo Horizonte MG, Brazil.

**Keywords:** Schizophrenia, Symptom Assessment, Cognition, Systematic Review, Esquizofrenia, Avaliação de Sintomas, Cognição, Revisão Sistemática

## Abstract

Schizophrenia is a complex psychiatric disorder characterized by positive and negative symptoms, as well as cognitive deficits, with an impact on functioning.

**Objective:**

To investigate the relationship between symptomatic remission, cognition, and functioning patients with schizophrenia, based on the criteria of the Remission in Schizophrenia Working Group, and to examine whether achieving remission reflects functional recovery.

**Methods:**

The search was conducted in the United States National Library of Medicine (PubMed) and *Biblioteca Virtual em Saúde* (BVS) databases, covering observational studies published between 2014 and 2023, in accordance with the Preferred Reporting Items for Systematic Reviews and Meta-Analyses (PRISMA).

**Results:**

After screening, 23 studies were included. The results indicated that remission is associated with better cognitive performance — such as memory, attention, processing speed, and flexibility — and functional outcomes.

**Conclusion:**

These findings reinforce the importance of consistent criteria for comparisons across studies and clinical practice. Remission is a desired outcome for many patients. However, given the current treatment resources, it remains an objective that only a limited number truly achieve. Investigating its influence is essential for developing more effective interventions.

## RESUMO.

A esquizofrenia é um transtorno psiquiátrico complexo, caracterizado por sintomas positivos, negativos e déficits cognitivos, com impacto na funcionalidade.

**Objetivo:**

Investigar a relação entre remissão, cognição e funcionalidade em pacientes com esquizofrenia, com base nos critérios do Remission in Schizophrenia Working Group (RSWG), e examinar se a remissão reflete a recuperação funcional.

**Métodos:**

A busca foi realizada nas bases Biblioteca Nacional de Medicina dos Estados Unidos (PubMed) e Biblioteca Virtual em Saúde (BVS), abrangendo estudos observacionais publicados entre 2014 e 2023, conforme o *Preferred Reporting Items for Systematic Reviews and Meta-Analyses* (PRISMA).

**Resultados:**

Foram incluídos 23 estudos após a triagem. Os resultados mostraram que a remissão está associada a melhor desempenho cognitivo — como memória, atenção, velocidade de processamento e flexibilidade — e funcional.

**Conclusão:**

Esses achados reforçam a importância de critérios consistentes para comparações entre estudos e a prática clínica. A remissão é um objetivo almejado para muitos pacientes, mas dados os recursos de tratamento atuais, continua sendo um objetivo que apenas alguns realmente alcançam. Destaca-se a necessidade de investigar sua influência para o desenvolvimento de intervenções mais eficazes.

## INTRODUCTION

 Schizophrenia is a complex and heterogeneous disorder with manifestations that vary from individual to individual^1-3^. It is thought to result from a combination of genetic, cerebral, hormonal, and environmental factors that are not yet fully understood^4,5^ and has a prevalence of approximately 1% in the population^6^. Patients with schizophrenia are diagnosed based on the manifestation of positive symptoms, but these are often accompanied by negative symptoms and cognitive impairments^7,8^, which in turn lead to functional impairments^9,10^. Cognition encompasses various mental abilities, including attention, memory, verbal fluency, and others, while functioning concerns an individual’s ability to perform social, occupational, and role-based activities in real-world contexts^3,8,9^. 

 For a long time, recovery or remission in schizophrenia was considered a rare outcome. However, with the advancement of psychotherapeutic techniques and antipsychotic medications, the discussion about the prognosis of the disease increased. An example of this is the consensus proposal for remission in schizophrenia developed by Remission in Schizophrenia Working Group (RSWG) according to a threshold in the severity of symptoms during a given period^11^. Through this consensus on remission, it would be possible to develop goals for the treatment of schizophrenia^12^, according to the criteria established by the Diagnostic and Statistical Manual of Mental Disorders^13^. In addition to the criteria proposed by the RSWG, there are other possibilities for assessing remission, such as using the Clinical Global Impression (CGI)^14^. Despite these alternatives, the remission criterion in schizophrenia proposed by the RSWG has proven to be conceptually viable, clinically valid and easy to use in clinical practice and research^13,15^. However, distinguishing symptomatic remission, which focuses on clinical symptoms, from functional recovery, which encompasses broader aspects such as cognition and social integration, is important. These outcomes are not necessarily aligned, and conflating them may obscure important treatment gaps^16-18^. Various studies have aimed to identify predictors of remission in schizophrenia^13,19-21^. 

 Despite advances in identifying predictors for schizophrenia, this has been a challenging task and only partially achieved in several studies^22-25^. Cognitive and functional aspects, in particular, have shown to be relevant indicators in the evaluation of remission, complementary to positive and negative symptoms^4,22,26,27^, given that the cognitive impairment of these patients, in relation to the healthy population, are well-documented^28^. Moreover, some biomarkers, such as inflammatory ones, have an impact on cognition and functioning, as well as schizophrenia symptoms, further emphasizing the importance of cognitive and functional aspects in the disorder^29-31^. 

 Nevertheless, cognition and functioning remain underexplored areas in the context of schizophrenia. RSWG consensus does not account for these two domains and the group recognizes the need for more research to better understand these areas, as already exists for other mental disorders^11,13,32^. 

 Although the RSWG has acknowledged the relevance of functional outcomes, its criteria were intentionally limited to the symptomatic dimension of the disorder^11^. However, symptomatic remission does not necessarily imply functional recovery, which entails the restoration of an individual’s capacity to engage meaningfully in social, occupational, and daily life roles^14,33,34^. Functional recovery encompasses the ability to maintain meaningful social relationships, engage in occupational roles, and achieve a level of autonomy compatible with community living^18,27,35^. Numerous studies have highlighted that patients who meet the criteria for symptomatic remission often continue to experience persistent cognitive deficits and significant impairments in real-world functioning^14,36-38^. As a result, there is growing consensus that recovery in schizophrenia should be understood as a multidimensional construct, with cognitive and functional domains playing a critical role in long-term outcomes^18^. 

 Therefore, cognitive and functional performance, although fundamental for assessing the recovery of patients with schizophrenia, are not included in the most commonly used remission criteria. This systematic review aimed to evaluate studies that approached the relationship between remission and cognitive and functional domains in individuals with schizophrenia, and to examine whether achieving remission reflects functional recovery.^39,40^. 

## METHODS

 This is a systematic review of the literature on the assessment of remission and cognitive and functional performance in patients with schizophrenia. For this purpose, articles were selected from the United States National Library of Medicine (PubMed) and *Biblioteca Virtual em Saúde* (BVS), that centralize access from Medical Literature Analysis and Retrieval System Online (Medline) and Latin American and Caribbean Health Sciences Literature (Lilacs) databases. The search employed the following terms: Schizophrenia[Title] AND Remission[Title] AND (cognit*[Title/Abstract] OR function*[Title/Abstract]). Articles published between 2014 and 2023 were selected, covering a period of ten years. The search strategy and selection of studies were carried out in accordance with the guidelines of the Preferred Reporting Items for Systematic Reviews and Meta-Analyses (PRISMA) 

 The inclusion criteria were: original and observational studies (longitudinal or cross-sectional) published in Portuguese or English, with patients diagnosed with schizophrenia. The focus on observational studies was due to the attempt to capture the natural state of remission without controlled interventions. The exclusion criteria were: clinical trials, review articles, comments, correspondence and those for which the full text was not available. 

 Once studies that allowed comparison between patients in remission and not in remission were identified, the following were considered: the remission criterion adopted, the forms of assessment and significant statistical associations (p<0.05). For this purpose, only the univariate analyses of the studies were considered, even if they described associations in multivariate analyses. Finally, individual tests were recorded independently, meaning that even if they belonged to a larger battery, only the specific test performed was considered. For example, the MATRICS Consensus Cognitive Battery (MCCB) includes tests from other batteries as the Letter Number Span (LNS) but this test was described individually. 

## RESULTS

**Figure 1:**
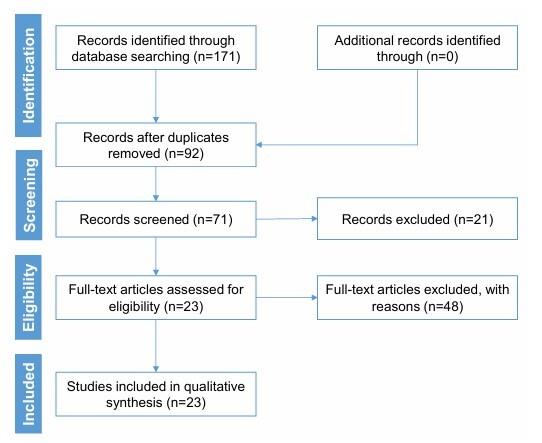
Flowchart of the study selection process for this review.

 In total, 171 articles were found in the selected electronic databases. Of these, duplicates (79) and full text not available (21) were excluded. Additionally, those that were not a study design compatible with the inclusion criteria or that did not fully approach the proposed theme (48) were also excluded. This left 23 articles eligible for this review. The flowchart of the selection process is presented in Figure 1. 

 Among the eligible articles that reported the study location, three were conducted in Turkey, two were multicenter, two in South Korea, two in India, two in Japan, and one each in the following countries: Germany, Brazil, China, Spain, Hungary, Italy, Mexico, Norway, Poland, Sweden, and Taiwan. Table 1 ^9,10,12,17,20,21,34,41-56^, presents the main characteristics of the studies included in this review. 

**Table 1:** List of the 23 articles included in this review and aspects related to remission in schizophrenia, according to the adoption of the criteria proposed by the Remission in Schizophrenia Working Group (RSWG).

Reference	Objective(s)	Cases (remitted)	Remission criteria (RSWG)
Kim et al.^12^	To determine whether mismatch negativity predicted remission six months later.	40 (19)	Yes
Jana^34^	To determine factors associated with remission from positive symptoms in Indian patients with schizophrenia.	151 (81)	Yes
Gorwood et al.^17^	(a) Correlate the target measure, i.e. FROGS-based functional remission, with three reference instruments to test the convergent validity of FROGS; (b) provide complementary empirical evidence to support this threshold thanks to external validators.	295 (97)	Yes
Sağlam et al.^10^	To compare remitted and symptomatic cases in terms of neurocognition and theory of mind.	72 (38)	Yes
Wang et al.^49^	To explore the association between symptomatic remission and employment outcomes, including the cumulative duration and income of work, with adjustments for related confounding variables in a two-year longitudinal study.	525 (124)	Yes
Ceylan et al.^44^	To compare cognitive dysfunction in bipolar disorder and schizophrenia during the remission and the symptomatic states.	80 (39)	Yes
Johansson et al.^9^	To explore which cognitive domains, including premorbid functioning, are related to the disorder prognosis in terms of sustainable remission.	173 (141)	Yes
Nakajima et al.^47^	To investigate the relationship between mismatch negativity and symptom remission in first-episode schizophrenia and their relationship with neurocognitive and social functions.	30 (14)	Yes
Ji et al.^21^	To determine whether self-report cognitive function is a predictor of symptomatic remission in amisulpride-treated schizophrenia.	303 (244/258* )	No
Correll et al.^41^	To assess the frequency of single and multiple concurrent dimensions of remission, as well as some basic demographic and illness characteristics as potential predictors of remission defined on the following three levels: remission of symptoms, functional remission and remission regarding subjective well-being.	194 (44)	Yes
Fond et al.^20^	To identify clinical and biological factors associated with impaired functional remission in nonselected chronic stabilized SZ outpatients.	273 (52)	Yes
Kim et al.^52^	To investigate the usefulness of peripheral inflammatory cytokine levels as a predictive biomarker of remission in patients with first-episode schizophrenia.	224 (174)	Yes
Madhivanan et al.^53^	To investigate whether the symptomatically remitted patients presented with better quality of life and social functioning compared to unremitted patients.	60 (30)	Yes
Kokaçya et al.^54^	To investigate functionality and quality of life in schizophrenia patients with symptomatic remission.	80 (40)	Yes
Yalınçetin et al.^51^	To examine the relation between FTD and SR in patients with schizophrenia. The other aim of this study is to identify the relation between FTD and social functioning.	117 (45)	Yes
Rabanea-Souza et al.^50^	To examine specific aspects of executive function of both remitted and nonremitted subjects, comparing with healthy controls.	114 (72)	Yes
Jaracz et al.^55^	To evaluate, longitudinally, the symptomatic remission in FE schizophrenic patients; to analyze psychopathological symptoms, social functioning and QoL in distinct patient groups selected on the basis of their symptomatic remission status; and to assess the 7–11-year outcome of the illness and to identify its predictors among baseline, pre-admission and early outcome variables.	64 (11)	Yes
Valencia et al.^56^	(a) To examine the rates of symptomatic remission, psychosocial remission, global functioning, and clinical global impression in a sample of schizophrenia outpatients; (b) to assess functional remission as the result of patients fulfilling the symptomatic, psychosocial remission and functioning criteria; (c) to identify predictive factors for functional remission; and (d) to compare assessments in different cultural settings with the results of the reported study.	168 (76)	Yes
Pinna et al.^48^	To evaluate the efficacy of a Clinical Global Impression-Schizophrenia (CGI-SCH) Severity score equal to or lower than 3 as a measure of remission in comparison to PANSS-based RSWG criteria.	112 (56)	Yes
Haro et al.^42^	To compare the quality of life of patients who achieve symptomatic remission of schizophrenia with those who do not achieve symptomatic remission.	6,516 †(2,476/2,932)	No
Torgalsbøen et al.^45^	To investigate the neurocognitive development in first-episode schizophrenia patients, and the influence of neurocognition on remission and real life functioning.	28 (17)	Yes
Fukumoto et al.^46^	To investigate the relations between remission status with considering time components and three cognitive functions of intellectual ability, memory and attention, which were examined before fulfilling the remission criteria, using longitudinal methods.	63 (33)	Yes
Balogh et al.^43^	To follow the changes of neurocognitive subfunctions during relapse and early remission (clinically stable state); in the present study patients with schizophrenia were tested in the acute phase and in clinically stable state, and then the results were correlated with clinical symptoms.	42 (-)	No

Notes: *- 244 (80.5%) achieved symptomatic remission by criterion A and 258 (85.1%) by criterion B;

†- 38% of patients were in symptomatic remission at the 12-month baseline visit. The percentage of patients in symptomatic remission increased to 45% at the 18-month visit and to 52% at the 36-month visit.

Abbreviations, FROGS, Functional Remission of General Schizophrenia; SZ, schizophrenia; FTD, Formal thought disorder; SR, Symptomatic remission; FES, first-episode schizophrenia; PANSS, Positive and Negative Syndrome Scale; RSWG, Remission in Schizophrenia Working Group.

 It was observed that approximately 87% of the studies included in this review adhered to the criteria proposed by the RSWG. Of these, only one study^41^ used the Brief Psychiatric Rating Scale (BPRS), while the others used the Positive and Negative Syndrome Scale (PANSS). Ji et al.^21^ used the PANSS to assess remission, but did not apply RSWG’s six-month minimum symptom severity criterion or two items from the general subscale (G5 and G9). Haro et al.^42^ assessed remission in schizophrenia using the Clinical Global Impression (CGI), while Balogh et al.^43^ considered two possibilities of remission, according to the PANSS: positive, calculated by the scores of the positive and general subscales; and negative, by those of the negative subscale. 

 Among the 23 studies identified in this review, 13 used tests or scales for cognitive assessment (Table 2)^9,10,12,21,43,44-47,48,49,50,51^. The majority (11) found an association between symptomatic remission and cognition. The scales varied across studies, with Mini-Mental State Examination (MMSE), identified in two studies as being associated with remission. In addition, Trail Making Test A (TMT-A) form was used in four studies, and in two of them^10,44^ an association with remission was identified, while in one study^9,45^ it was not identified. The TMT-B form was used in only three of these four studies and was associated with remission only in one study^10^. The Wechsler Adult Intelligence Scale (WAIS) was used in three studies, with processing speed and vocabulary associated with remission in two: Johansson et al.^9^ and Fukumoto et al.^46^. 

**Table 2:** List of studies included in this review that reported the use of cognitive assessment scales (n=13).

Author(s) and year	Cognitive assessment scales	Remission vs. cognition
Kim et al.^12^	WAIS – intelligence quotient	No
Sağlam et al.^10^	WISC	No
	RAVLT – total scores of verbal learning	No
	RAVLT – late recall	No
	RAVLT – true recognition	No
	WMS – immediate recall	No
	WMS – late recall	No
	Semantic fluency/animals	No
	COWAT	No
	ST	No
	ST – interference	No
	TMT – A form	Yes
	TMT – B form	Yes
	WISC – digit-symbol	Yes
	ACTT	No
	RMET	Yes
	Faux Pas Test	No
Wang et al.^49^	MMSE	Yes
Ceylan et al.^44^	RAVLT – verbal learning (trial 1–5)	No
	RAVLT – delayed recall (trial 7)	No
	RAVLT – total recognition (true recognition)	No
	VRT – immediate recall	No
	VRT – delayed recall	No
	DST – backward	Yes
	ACTT	No
	STD	Yes
	WCST – category	Yes
	WCST – perseveration	No
	ST – interference	No
	TMT – A form	Yes
	TMT – B form	No
	COWAT	Yes
	Animal Naming Test	Yes
Johansson et al.^9^	CPT – full prime	No
	TMT – A form	No
	TMT-B form	No
	RAVLT – A1-A5	No
	RAVLT – A7	No
	LNS	Yes
	WAIS – Vocabulary	Yes
	WCST – category	No
Nakajima et al.^47^	BACS	No
	SCORS	Yes
Ji et al.^21^	PDQ	Yes
Yalınçetin et al.^51^	TLI	Yes
Rabanea-Souza et al.^50^	Nonverbal Intelligence Task (R-1)	No
	Computerized Stroop Test	Yes
	Keep Track Task	Yes
	Letter Memory Task	Yes
	Number-Letter Task	No
	Plus-Minus Task	No
	Tower of London Test	No
Pinna et al.^48^	BACS – list learning	No
	BACS – digit sequencing task	Yes
	BACS – verbal fluency/category instances	No
	MMSE	Yes
Torgalsbøen et al.^45^	TMT – A form	No
	BACS – Symbol Coding	No
	HVLT-R	Yes
	WMS – Spatial Span	No
	LNS	No
	BMVT-R	No
	NAB	No
	MSCEIT	No
	CPT-IP	Yes
Fukumoto et al.^46^	WAIS – Full scale IQ	No
	WAIS – Verbal IQ	No
	WAIS – Performance IQ	No
	WAIS – Verbal Comprehension	No
	WAIS – Perceptual Organization	No
	WAIS – Working Memory	No
	WAIS – Processing Speed	Yes
	WMS – Verbal Memory	No
	WMS – Visual Memory	No
	WMS – General Memory	No
	WMS – Attention / Concentration	Yes
	WMS – Delayed Recall	No
	CPT – Digit2 D’	Yes
	CPT – Digit3 D’	Yes
	CPT – Digit4 D’	No
Balogh et al.^43^	CANTAB – Paired Associate Learning	No
	CANTAB – Spatial Recognition Memory	No
	CANTAB – Rapid Visual Information Processing	No
	CANTAB – Spatial Working Memory	No
	CANTAB – Stockings of Cambridge	No

Abbreviations, ACT, Auditory Consonant Trigrams Test; BACS, Brief Assessment of Cognition in Schizophrenia; BMVT-R, The revised Brief Visuospatial Memory Test; CANTAB, Neuropsychological Test Automated Battery; COWAT, Controlled Oral Word Association Test; CPT-IP, Continuous Performance Test, Identical Pairs; DST, Digit Span Test; GF, Global Functioning; HVLT-R, Hopkins Verbal Learning Test; LNS, Letter-Number-Sequencing; MCCB, MATRICS Consensus Cognitive Battery; MMSE, Mini-Mental State Examination; MSCEIT, Mayer Salovey Caruso Emotional Intelligence Test; NAB, Neuropsychological Assessment Battery; PDQ, Perceived Deficits Questionnaire; RAVLT, Rey Auditory Verbal Learning Test; RMET, Reading the Mind in the Eyes Test; SCORS, Schizophrenia Cognition Rating Scale; ST, Stroop Task; TLI, Thought and Language Index; TMT, Trail Making Test; VRT, Visual Reproduction Test; WAIS, Wechsler Adult Intelligence Scale; WCST, Wisconsin Card Sorting Test; WISC, Wechsler Intelligence Scale for Children; WMS, Wechsler Memory Scale.

 Other cognitive assessment tools that identified associations with remission included: Animal Naming Test; digit sequencing task from the Brief Assessment of Cognition in Schizophrenia; Computerized Stroop Test; Continuous Performance Test — Identical Pairs (CPT-IP), and the subtests Digit2 D’ and Digit3 D’; the Digit Span Test (DST), general and in the backward subtest; Hopkins Verbal Learning Test (HVLT-R); Keep Track Task; LNS; Perceived Deficits Questionnaire (PDQ); Reading the Mind in the Eyes Test (RMET); Schizophrenia Cognition Rating Scale (SCORS); Thought and Language Index (TLI); digit symbol of Wechsler Intelligence Scale for Children (WISC); and the Wechsler Memory Scale (WMS). 

 When the Controlled Oral Word Association Test (COWAT) was used, Ceylan et al.^44^ found a statistically significant association with remission, while Sağlam et al.^10^ did not. The category subscale of the Wisconsin Card Sorting Test (WCST) was also associated with remission in one study^44^, but not in another^9^. 

 Other tests or scales, such as Faux Pas Test and Auditory Consonant Trigrams Test, used for cognitive assessment, did not find an association between symptomatic remission and cognition. 

 Of the 17 studies that used some functional scale to assess symptomatic remission, 15 identified a significant statistical association across these two dimensions (Table 3)^10,12,17,20,34,41,42,46-49,51-56^. Among the functional assessment scales, the most used were the Global Assessment of Functioning (GAF), in ten studies, and the Personal and Social Performance Scale (PSP), in six studies. In all studies that used these two scales, an association between remission and functioning was identified. In addition, an association between functioning and symptomatic remission was identified using Functional Remission of General Schizophrenia (FROGS), Independent Living Skills Survey (ILSS), Psychosocial Remission Scale (PSRS) and Social Functioning Scale (SFS). One study identified an association between symptomatic remission and functioning using the Premorbid Adjustment Scale (PAS)^34^, while another did not^10^, regardless of the academic or social subscale. 

**Table 3:** List of studies included in this review that reported the use of functional assessment scales (n=17).

Reference	Functional assessment scales	Remission vs. functioning
Kim et al.^12^	GAF	Yes
Jana^34^	GAF	Yes
	PAS	Yes
	ILSS	Yes
Gorwood et al.^17^	FROGS	Yes
	GAF	No
	PSP	Yes
	PSRS	Yes
Sağlam et al.^10^	PAS	No
	PAS – Social	No
	PAS – Academic	No
Wang et al.^51^	PSP	Yes
Nakajima et al.^47^	GAF	Yes
Correll et al.^41^	GAF	Yes
Fond et al.^20^	GAF	Yes
Kim et al.^52^	PSP	Yes
Madhivanan et al.^53^	PSP	Yes
	GAF	Yes
Kokaçya et al.^54^	GAF	Yes
Yalınçetin et al.^51^	PSP	Yes
Jaracz et al.^55^	SFS	Yes
Valencia et al.^58^	GAF	Yes
Pinna et al.^48^	PSP	Yes
Haro et al.^42^	Own criteria	Yes
Fukumoto et al.^46^	GAF	Yes

Abbreviations, FROGS, Functional Remission of General Schizophrenia; GAF, Global Assessment of Functioning; ILSS, Independent Living Skills Survey; PAS, Premorbid Adjustment Scale; PSP, Personal and Social Performance; PSRS, Psychosocial Remission Scale; SFS, Social Functioning Scale.

## DISCUSSION

 The findings of this review support the evidence that cognition and functioning are associated with symptomatic remission in patients with schizophrenia^12,21,47^. Cognition, in particular, has been widely recognized as a relevant predictor of patients’ functioning and quality of life^4^. Despite this recognition, most current treatment approaches continue to prioritize the remission of positive symptoms, often overlooking the broader goal of functional recovery. This gap underscores the importance of research that targets cognitive and functional outcomes as essential components of recovery, beyond symptomatic control^6,9,38,57^. 

 Most of the studies included in this review adhered to the criteria established by the RSWG. The early applications of the group’s proposal favor the identification of signs of symptom severity from the first crisis to the evaluation of treatment efficacy over time^13,15^. 

 The tests and scales used in the studies included in this review, which identified an association between cognition and symptomatic remission, assess several cognitive functions. The MMSE was commonly employed to evaluate global cognitive function^48,49,58^. Although not primarily designed for this purpose, DST and WMS can also assess attention and concentration, in addition to their primary function of evaluating memory, particularly verbal memory^44,46,59,60^. Processing speed was assessed by the WAIS^46,61,62^, by the digit symbol of WISC^10,61,62^, by Animal Naming Test^44,59^ and by the TMT^10,44,63,64^, which also measured inhibitory response^44,63^. Working memory is measured by LNS^9,61^, by Digit sequencing task of the BACS^48,65^ and Keep Track Task^50,66^. Verbal memory was examined by HVLT-R^45,59^ and vocabulary subscale of WAIS^9,54,61,62^. Attention and vigilance were assessed by variations of the CPT-IP^45,46,67^. Inhibitory control and cognitive flexibility were assessed by the Computerized Stroop Test^50^ and TMT^10,44,63,64^. Verbal fluency was assessed by the Animal Naming Test^44,59^ and TLI^51,68^. Social cognition was investigated by RMET^10,69^. Perceived cognitive deficits were measured using PDQ^21,70^ and SCORS^47,71^. 

 Some of the tests associated with remission, as identified in this review, are included in batteries specifically designed for patients with schizophrenia. This includes MCCB and BACS. The MCCB assesses cognitive domains such as processing speed (Trail Making Test Part A, BACS subtests Symbol Coding, and Animal Naming), vigilance/attention (CPT-IP), working memory (WMSIII), verbal learning/memory (HVLT-R), visual learning/memory (BVMT-R), reasoning/problem solving (Neuropsychological Assessment Battery), and social cognition (MSCEIT)^59,72^. The BACS measures verbal memory, working memory, motor speed, attention, executive function, and verbal fluency, using tests such as the WAIS subtests, Wechsler Memory Scale, and Wisconsin Card Sorting Test^73^. 

 A systematic review^74^ conducted between 1995 and 2006 highlighted neuropsychological tests frequently used in schizophrenia. Among the most cited tests in the 1995 and 2006 review and those observed in this review are WCST, WAIS, TMT, ST and WMS. The same tests can evaluate more than one function and are renowned in scientific and professional literature, which makes it easier to choose these instruments. 

 Functioning was also significantly associated with remission in all studies that evaluated this relationship. Instruments such as the GAF^75^ and PSP^76^, although not specific to schizophrenia, have proven useful in assessing patients’ global functioning. The PSRS is dedicated to schizophrenia, but measures psychosocial remission, which is only a part of functional remission, and also includes items that overlap with symptomatology. The FROGS has the ability to reflect the negative and cognitive aspects of schizophrenia^17,77^. The ILSS^78^ is composed of nine subdomains that focus on and describe specific issues relevant to real-world functioning. In addition to these, an association was identified between symptomatic remission and performance on the SFS^79^ and PAS^34^. Measurements of daily functioning have focused on the endpoint of real-world performance in an attempt to classify the level of disability based on daily activities performed inadequately or not performed at all. Each of these instruments has distinct objectives and assessment criteria, although they have proven to be reliable indicators in assessing functioning^17,27^. 

 Although functional outcomes are strongly correlated with symptomatic remission and better subjective life satisfaction in patients with schizophrenia, they do not necessarily equate to functional remission across all domains^16^. Assessment of the social functioning of patients with schizophrenia is important for understanding the social impact of this disorder and for assessing the influence of pharmacological and/or psychosocial interventions on the social performance of patients^80^. 

 Although this review identified a clear relationship between remission, cognition, and functioning, some limitations must be considered. The included studies differed in their objectives and methods, such as different designs and patient follow-up time, preventing standardization of findings. In addition, the sample was heterogeneous, with studies involving patients ranging from first-episode to hospitalized patients. These methodological variations limit the generalization of results. Despite these limitations, reviews such as this one contribute to the process of standardizing criteria and facilitating comparisons between studies. 

 In conclusion, most studies identified in this review reported a relationship between remission and cognition or functioning in schizophrenia. Although the studies are not homogeneous, remission has been shown to be a viable state for many individuals with schizophrenia and the cognitive and functional aspects accessible to generate more specific evidence that contributes to advanced research in the field. Moreover, the cognitive and functional aspects of remission are frequently undervalued in clinical assessments and decision-making processes, in part because these evaluations often fall under the expertise of non-physician professionals^18^. This fragmentation highlights the need for public health policies that promote truly interdisciplinary approaches to mental health care, ensuring that the contributions of psychologists, occupational therapists, and social workers are integrated into routine psychiatric evaluations. 

## Data Availability

No new data were generated or analyzed in this study.
